# Juvenile psammomatoid ossifying fibroma of Orbit-A rare case report and review of literature

**DOI:** 10.4317/jced.57897

**Published:** 2021-06-01

**Authors:** Arunkumar Kamalakaran, Bharathi Ramakrishnan, Rohini Thirunavukkarasu

**Affiliations:** 1 Associate Professor, Department of Oral and Maxillofacial Surgery, Tamilnadu Government Dental College and Hospital, Chennai; 2 Professor, Department of Oral and Maxilofacial Pathology, Tamilnadu Government Dental College and Hospital, Chennai; 3 Senior Assistant professor, Department Of Oral and Maxillofacial Surgery, Tamilnadu Government Dental College and Hospital, Chennai

## Abstract

Fibro osseous lesions of the craniofacial skeleton are a benign condition in which the normal architecture of the bone is replaced by fibrous connective tissue with varying degrees of mineralization. JOF forms a special entity among the fibro osseous lesions because of its age of occurrence and its aggressive nature thereby mimicking a malignancy. The Juvenile Ossifying Fibromas were further subdivided into Psammomatoid and Trabecular variant based on their histopathological characteristics. They tend to differ in their mineralized portion with the trabecular variant showing woven bone while the psammamotoid shows lamellated and spherical ossicles in various shapes in a myxoid stroma intermingled with bone cyst like areas. The reported cases of JPSOF are few, hence histopathological examination is a valuable tool in the diagnosis of this rare lesion and JOF should always be considered in the differential diagnosis of the lesions of the craniofacial skeleton. Early diagnosis and a complete surgical excision with adequate margins and a long term follow up is mandatory for a good prognosis of this highly recurrent and aggressive lesion.The diagnosis of JOF requires a careful correlation of clinical, radiological and histopathological features. The purpose of this paper is to report a case of JPOF of the orbit to stress the need for consideration of JOF in the differential diagnosis of the aggressive lesions of the Cranio facial skeleton.

** Key words:**Juvenile psammomatoid ossifying fibroma, orbit, ossifying fibroma.

## Introduction

Fibro osseous lesions of the craniofacial skeleton are a benign condition in which the normal architecture of the bone is replaced by fibrous connective tissue with varying degrees of mineralization. They are broadly classified as cemento-osseous dysplasia,Ossifying fibroma and Fibrous Dysplasia. Ossifying fibromas are in turn further divided into Conventional(Cemento-Ossifying fibroma) and Juvenile Ossifying Fibroma( JOF) (1).

The term Juvenile active Ossifying fibroma was first coined by Golg (2) in 1949. JOF forms a special entity among the fibro osseous lesions because of its age of occurrence and its aggressive nature thereby mimicking a malignancy. The clinical features of JOF are mainly asymmetric expansion of the affected jaws leading to facial asymmetry. Pain and paraesthesia are a rare entity inspite of the aggressive nature of these lesions. Complications such as nasal obstruction,epistaxis,exophthalmos and intracranial extensions are encounterd only in aggressive tumors involving the paranasal sinuses,orbit and Maxilla. Radiographically they appear as unilocular or multilocular radiolucency with well defined sclerotic borders and central opacification depending on the extent of calcification. Aggressive tumors however show signs of cortical thinning and perforation.The management of JOF involves complete surgical excision.

The Juvenile Ossifying Fibromas were further subdivided into Psammomatoid and Trabecular variant based on their histopathological characteristics according to the WHO classification of odontogenic tumors 2005 (3). Histologically, loose fibroelastic tissue with areas of collagen deposition leading to mineral deposition resulting in the formation of woven bone with trabecular pattern are seen in Juvenile Trabecular Ossifying fibroma whereas in case of Juvenile Psammomatoid Ossifying fibroma, cellular fibrous stroma with characteristic spheroidal calcifications called Psammoma bodies are seen (4).

El Mofty *et al*. (5) reported that apart from the histopathological chraracteristics there seemed to be a significant demographic difference between the two variants of JOF,they observed that the trabecular variant occurs most commonly in the jaws with a maxillary predilection with the average age of occurrence being eight and a half years and on the contrary, the psammoma variant was found most commonly to occur in the sinonasal and orbital bones with the average age occurrence being in the wider age range of 16-33 years. The incidence of JOF is not known till date because of the very few number of cases reported. The diagnosis of JOF requires a careful correlation of clinical,radiological and histopathological features. The purpose of this paper is to report a case of JPOF of the orbit to stress the need for consideration of JOF in the differential diagnosis of the aggressive lesions of the Cranio facial skeleton.

## Case Report

**Figure 1:**
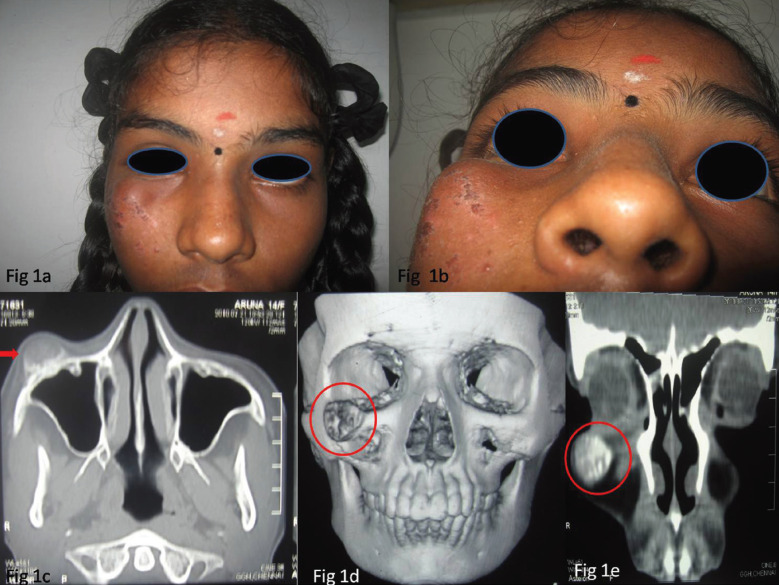
a- Profile view of the patient showing Extraoral swelling. b- Extra oral view showing ulcerated skin. c- Computed Tomography(CT) Axial View showing lesion of mixed densities in the infraorbital rim. d-CT -3D reconstruction image showing lesion of mixed densities in the infraorbital rim. e-CT-coronal view showing lesion of mixed densities in the infraorbital rim.

A 15 year old female patient presented with a chief complaint of painless swelling below the right eye since six months. History revealed that the swelling was slowly progressive and attained the present size gradually over a period of six months. There was no significant past medical or surgical history. On Extra oral examination a swelling of size 4×3cm was seen extending from the inferior border of the orbit superiorly till the roof of maxillary sinus inferiorly,medially it extended till the lateral nasal wall and laterally till the body of the Zygoma (Fig. 1a). Overlying skin was thinned out and ulcerated in certain areas (Fig. 1b) and on palpation the swelling was firm in consistency and non tender and the skin overlying the swelling was pinchable. Opthmalogic examination showed no signs of proptosis,exophthalmous or progressive loss of vision. The vision and the extra ocular movements were normal. There was a mild facial asymmetry seen. Introral examination revealed no evident findings. There were no evidence of maxillary or infraorbital nerve paraesthesia.

The computed tomography CT scan of the patient demonstrated a well circumscribe lesion with varying densities (both hyper and hypodense) of size 2×2cm involving the infraorbital rim not involving the maxillary sinus or the nasal septum (Fig. 1c-e). A provisional diagnosis of ossifying fibroma was made based on the clinical and the radiological findings. An excisional biopsy was planned taking into consideration the location and size of the lesion.

**Figure 2:**
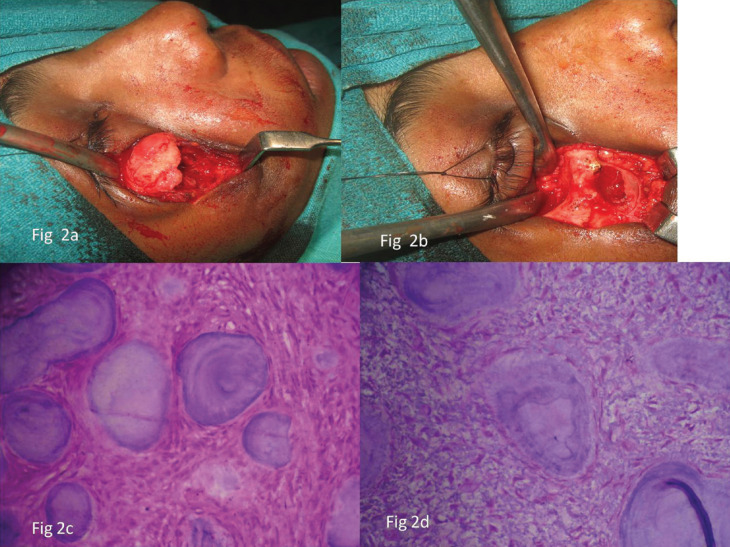
a- Exposure of the Lesion. b- Excision of the lesion and Peripheral ostectomy. c- Photomicrograph showing ossicles resembling psamama bodies in a fibrocellular stroma (high power ,H&E stained×10). d- Photomicrograph showing ossicles resembling psamama bodies in a fibrocellular stroma (H&E stained×40).

**Figure 3:**
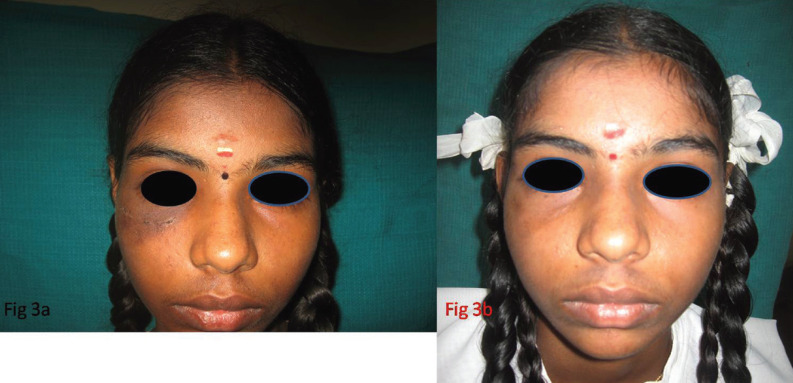
a-Profile view of the patient immediate post op. b- Profile view of the patient after a 5 year follow up.

The surgical procedure was done under general anaesthesia using naso endotracheal intubation. A infraorbital incision was given, periosteum was incised and the lesion was identified and excised and peripheral ostectomy was done (Fig. 2a,b). The wound was closed using – vicryl and prolene layerwise. Post operatively the specimen was sent for histopathological examination and the report revealed that numerous spherical calcifications were seen in a fibrocellular stroma and concentric lamellated calcifications resembling psammoma bodies were seen within the stroma suggestive of Psammomatoid Juvenile Ossifying Fibroma (Fig. 2c,d). The patient was followed up monthly for 6 months and 6 months once postoperatively till date and there has been no evidence of any recurrence (Fig. 3a,b).

## Discussion

Juvenile aggressive ossifying fibroma is a benign fibro osseous lesions of jaws seen occurring predominantly in children and adolscents. According to WHO it is describred as a lesion affecting individuals below the age of fifteen (6). Juvenile aggressive ossifying fibroma is divided into psammamotous and trabecular pattern. They tend to differ in their mineralized portion with the trabecular variant showing woven bone while the psammamotous shows lamellated and spherical ossicles in various shapes in a myxoid stroma intermingled with bone cyst like areas (7). 

El- Mofty (5) reported in their literature review that the JPSOF occurs in older and in a wider age group. The mean age of occurrence JPSOF is approximately 20 years whereas in case of JTOF, the mean age of occurence is 81/2 to 12 years suggesting JTOF is more common in children and adolescents. In the current case the patient was 15 yrs considering the age of the patient it is more in favour of JTOF however literature review (8,9) reports that JPSOF have been observed in patients as young as three months and as old as seventy two years and the present case is a classic example of this wide age range of JPSOF as the patient was 15yrs which is the age more common for JTOF.

JPOF mainly involves the bone of the orbit and the paranasal sinuses , on the contrary JTOF involves the maxilla or mandible although the jaw predilection seems to be controversial (10). In our case the location of the lesion was in the infraorbital region which adds to the diagnosis of JPSOF. Makek *et al*. (11) in his review of 86 cases of JPSOF reported a mean age of occurrence of 17.7yrs with slight male predominance and Orbital bones and Paranasal sinuses were the most common site (61.6%). Similar observations of male predilection and highest occurrence of 70% of cases in the paranasal sinuses was reported by Johnson *et al*. (3) in their review of 112 cases of JPSOF. However in Slootwegs (13) review of 23 cases 16 cases were found in the mandible, followed by the maxilla (6) and Paranasal sinuses (2).

Margo *et al*. (14) divided the orbital involvement in JPSOF into two subgroups based on their location as supraorbital involving the orbital rim and the frontal sinus and the other group was of ethmoid sinus origin. They observed that the orbital involvement was never solitary and always occurs along with involvement of the frontal or the ethmoid sinuses. They concluded that orbital involvement and the ophthmalogic signs and symptoms noted are always secondary and it is not the representative primary site. In total contrast to this, in the reported case there was a isolated lesion in the infraorbital rim without any other paranasal sinuses involvement.

The most common clinical feature of JPSOF is proptosis due to pressure effect of the lesion,lateral displacement of the eye ball have also been noticed in some cases. In aggressive and extensive lesions decreasing vision with progressive blindness have also been reported (15). Nasal obstruction, headaches ,facial swelling, pain, recurrent sinusitis ranging from several weeks to months,missing teeth are other clinical findings observed in JPSOF. The pattern of local invasion varies from “bowing ’’ or “pushing’’ of the adjacent bony walls to invasion through the adjacent bone leading to extension to other adjacent anatomic structures. Some studies (16) state that the aggressive growth and recurrence tendency are age related and is seen more frequently in younger age groups. In case of jaw involvement painless asymmetric swelling with expansion of the cortical plates are seen. Facial swelling with asymmetry, pain were the only clinical manifestations observed in the current case.

Radiographically the lesions can be radiolucent, mixed or radioopaque depending on the extent of calcification resembling other fibro-osseous lesions such as fibrous dysplasia and cement ossifying fibroma (17). PSOF may cause expansion and perforation of the cortex because of the huge size and aggressive nature. There was no evidence of cortical perforation but a mild cortical expansion was observed in the reported case. The lack of perforation may be attributed to a earlier diagnosis and management of the disease.The typical radiographic feature which distinguishes both the variants of JOF from the fibro-osseous lesions is that its margins are well defined and do not blend with the adjacent bone. This characteristic feature was evident in our case also adding to the diagnosis of JOF. Owosho *et al*. (18) reported that both JTOF and JPSOF displayed well defined borders although JPOF exhibited a ground glass pattern as an outer mantle with central radiolucency or a single mural nodule or a solid homogenous mass. On the contrary, JTOF appears as a radiolucent lesion with irregular scattered calcifications.

Radiographically the differential diagnosis of JPSOF include malignant lesions such as osteosarcoma,chondrosarcoma, Ewing’s sarcoma, African form of Burkits lymphoma, benign lesions such as conventional Ossifying Fibroma, Fibrous Dysplasia and mixed odontogenic tumors (19).

Aneurysymal bone cysts have been observed in association with JOF (20). They develop initially as a focal myxoid change in the stroma with haemorrhage and osteoclastic giant cells leading to gradual expansion and formation of cysts with their fibrous wall. JOF associated with aneurysmal bone cyst are more common in younger patients and in large aggressive maxillary lesions. Chrcanovic *et al*. (21) reported that JOF associated with aneurysmal bone cyst are more prevalent in JPOF compared to JTOF and lesions with ABC had higher rate of recurrence compared to JOF without cysts (22).

Johnson *et al*. (12) reported that JPSOF occurred as result of excessive production of the myxofibrous cellular stroma involved in the growth of the paranasal sinuses septa as the sinuses enlarge and pneumatise during growth. The hyaline material secreted by the stromal cells ossifies and the connective tissue mucin intiates the cystic areas resulting in JPSOF. *Pi*menta *et al*. (23) stated that mutations in HRPT2 genes leading to haploinsufficiency of HRPT2gene are found in OF. In contrast such genetic alterations are absent in JOF suggesting that it could be a different clinical entity ,however they concluded that further studies are needed to confirm this. The presence of translocation by nonrandom chromosomal breakpoints at Xq26 and 2q33 and disturbance in the basal generative mechanism which is a prerequisite for root formation have also been reported to be the possible pathogenesis of JOF (24,25).

Histologically JPSOF are characterized by the presence of concentric ossicles called psammoma bodies (26). These psammomatoid ossicles contain osteocytes indicating a osteogenic origin. The ossicles vary from small with round to oval shape to large and irregular shape. Similar findings were noted histologically in the current case. The histopathological differential diagnosis of JPSOF include cementifying fibromas,cementoblastoma and familial gigantiform cementoma since the cementicle resemble psammomatoid spheicles. The cementicles however are characterized by an acellular matrix without associated osteocytes, basophilic appearing reversal or cement lines,an eosinophilic fibrillar appearance with a dark staining periphery and finer or more delicate parallel birefringent lines than the ones in lamellar bone under polarized light. These features help to distinguish JPSOF from the cementum forming lesions (27). Moreever, cementum does not show immunoreactivity with osteonectin which is a bone- specific protein or the ultra structural features suggestive of osteogenic origin. Immunohistochemical studies (28,29) on JPSOF with osteonectin showed reactive spindle cell component and osteoid suggesting osseous origin. The aggressive nature has been attributed to presence of CK but Williams *et al*. (30) did not find CK expression in 8 cases of JPOF. Low Ki-67/MIB-1 proliferation index was observed by Hasselblatt *et al*. (31). Immunohistochemical findings similar to cement- ossifying fibroma arising from the tooth bearing areas and meningiomas such as expression of vimentin,EMA,SMA and CD10 with absence of expression of CD34,S-100 protein and cytokeratins has been reported by Granados *et al*. (32), but EMA negative cases have also been reported in the literature (33). However immunohistochemistry was not done in the present case.

Primary extracranial psammomatoid meninigioma (PEPM) presents a major diagnostic challenge in cases of JPSOF in paranasal sinuses because the clinical and radiographic features are very similar (34). They can be differentiated only through histopathological examination wherein the regular shape of the psammoma bodies and the concentric lamellar pattern of calcospherites seen in JPSOF are not seen in PEPM. In addition , the psammoma bodies are hapazhardly distributed in PEPM whereas in JPOF they are homogenously distributed in the stroma.

The management of JOF ranges from complete surgical resection ,enucleation and curettage and enucleation with peripheral ostectomy (35). Smaller lesions can be treated by enucleation and curettage whereas larger lesions need resection with 5mm margins. Resection should be considered in cases of recurrent,aggressive lesions or where inferior border of the mandible cannot be preserved. Preservation of the periosteum is prudent for regeneration of bone as it has osteogenic potential and to prevent soft tissue prolapse. Though some sinonasal cases have been treated endoscopically (36) open surgical approach is needed for adequate visibility. The surgical approach and the treatment modality depends on the location,extent and the relationship of the lesion to adjacent anatomic structures. Taking into consideration all these factors enucleation and peripheral ostectomy was done in the reported case.

The recurrence rate of JPOF is reported to be 30% to 56% (37). The common reasons for recurrence are incomplete resection resulting in residual disease, spillage of the tumor cells during resection and in case of long standing lesions extensive cortical destruction and periosteal elevation increases the risk of recurrence. It has been reported that the recurrence occurs from 6 months to 19 years after surgical excision of the lesion. There has been no evidence of recurrence in the current case after a five year follow up.

## Conclusions

Juvenile Ossifying fibroma is a benign fibro osseous lesion with a high recurrence rate and aggressive behavior clinically mimicking a malignancy. Hence a proper diagnosis based on a complete clinical,radiological and histopathological examination is warranted to avoid a misdiagnosis and for executing the correct treatment modality. The reported cases of JPSOF are few, hence histopathological examination is a valuable tool in the diagnosis of this rare lesion and JOF should always be considered in the differential diagnosis of the lesions of the craniofacial skeleton. Early diagnosis and a complete surgical excision with adequate margins and a long term follow up is mandatory for a good prognosis of this highly recurrent and aggressive lesion.
